# Identification of post-COVID-19 condition phenotypes, and differences in health-related quality of life and healthcare use: a cluster analysis

**DOI:** 10.1017/S0950268823001139

**Published:** 2023-07-18

**Authors:** Iris Gerritzen, Iris M. Brus, Inge Spronk, Sara Biere-Rafi, Suzanne Polinder, Juanita A. Haagsma

**Affiliations:** 1 Department of Public Health, Erasmus University Medical Center, Rotterdam, The Netherlands; 2 C-support, ‘s Hertogenbosch, The Netherlands

**Keywords:** COVID-19, health related quality of life, Post COVID-19 condition, healthcare use, cluster analysis

## Abstract

The aim of this cross-sectional study was to identify post-COVID-19 condition (PCC) phenotypes and to investigate the health-related quality of life (HRQoL) and healthcare use per phenotype. We administered a questionnaire to a cohort of PCC patients that included items on socio-demographics, medical characteristics, health symptoms, healthcare use, and the EQ-5D-5L. A principal component analysis (PCA) of PCC symptoms was performed to identify symptom patterns. K-means clustering was used to identify phenotypes. In total, 8630 participants completed the survey. The median number of symptoms was 18, with the top 3 being fatigue, concentration problems, and decreased physical condition. Eight symptom patterns and three phenotypes were identified. Phenotype 1 comprised participants with a lower-than-average number of symptoms, phenotype 2 with an average number of symptoms, and phenotype 3 with a higher-than-average number of symptoms. Compared to participants in phenotypes 1 and 2, those in phenotype 3 consulted significantly more healthcare providers (median 4, 6, and 7, respectively, *p* < 0.001) and had a significantly worse HRQoL (*p* < 0.001). In conclusion, number of symptoms rather than type of symptom was the driver in the identification of PCC phenotypes. Experiencing a higher number of symptoms is associated with a lower HRQoL and more healthcare use.

## Introduction

Globally, there have been more than 645 million confirmed cases of COVID-19 registered since the start of the pandemic [1]. An estimated 4–12% of patients experience long-term symptoms after infection with SARS-CoV-2; this is referred to as long COVID or post-COVID-19 condition (PCC) [2, 3]. PCC is defined by the World Health Organization (WHO) as ‘*the continuation or development of new symptoms 3 months after the initial SARS-CoV-2 infection, with these symptoms lasting for at least 2 months with no other explanation’* [4]. PCC can be prevalent after both symptomatic and asymptomatic COVID-19 infections [5, 6]. The global health burden of PCC is growing [7], as well as its associated costs, and is estimated to have a prevalence of over 144 million individuals affected to date [8].

Those with PCC generally experience a wide variety of symptoms [9], with the clinical manifestation differing among patients [10]. Frequently reported symptoms include shortness of breath, fatigue, increased heart rate, and cognitive problems [9]; however, patients can experience many other symptoms that vary in severity [11, 12]. The multitude of symptoms reported by patients involve different organ systems [13], and the underlying mechanisms as to why patients experience long-term symptoms after COVID-19 are still unknown [14]. However, several hypotheses are made including immune dysregulation, microbiota disruption, autoimmunity and immune priming, blood clotting and endothelial abnormalities, and dysfunctional neurological signalling [15]. PCC symptoms can last for months or years, and it is unclear whether they disappear [16]. In addition to physical symptoms, PCC affects mental well-being, with higher rates of mood disorders and anxiety disorders being reported in patients with PCC than in patients with other respiratory tract infections or influenza [17]. The impact on mental well-being can only partially be explained by the disruptiveness and unpredictability of the pandemic and preventive and protective measures against the spread of COVID-19, such as quarantine and social distancing [18]. The acute and post-acute neuropsychiatric sequelae seen in patients with COVID-19 also profoundly impact mental well-being [18, 19]. Furthermore, long-term symptoms of COVID-19 negatively impact the health-related quality of life (HRQoL) [20, 21]. Meta-analysis of PCC in relation to the HRQoL shows that 58% of patients report a poor HRQoL on the EQ-5D-5L [20]. This systematic review however does not distinguish between subgroups, differences in symptoms, or severity.

Management and care for PCC is posing a substantial burden on healthcare systems [22, 23]. The knowledge of long-term effects of COVID-19 and treatment options for PCC are still evolving [24]. Due to the nature of PCC, treatment often requires a multi-disciplinary approach that includes a thorough evaluation, treatment of symptoms, treatment of underlying problems, physical therapy, occupational therapy, and psychological support [24, 25]. Identifying subtypes of PCC might help tailor treatment approaches by allowing treatment plans to be specified to the symptoms experienced in each subtype, and including only relevant healthcare providers for those specific symptoms in the treatment plan. It can help healthcare systems, healthcare providers, and patients to be better prepared and informed in managing PCC. The existing literature on PCC symptom clusters or subtypes mainly focuses on symptom patterns, frequently reported symptoms, demographic and medical background characteristics, and potential risk factors [10, 26–29]. Furthermore, Deep Phenotyping by Human Phenotype Ontology (HPO) has been applied to PCC to map the phenotypic profile of PCC [10]. This study aimed to add to the existing literature by not only identifying PCC phenotypes but also investigating healthcare use and the HRQoL per phenotype identified.

## Methods

### Study design and population

In this cross-sectional study, questionnaire data from patients with PCC were used. This study was conducted in collaboration with C-support, a Dutch organisation that informs, advices, supports, and provides care for patients experiencing long-term symptoms after SARS-CoV-2 infection. Patients can ask for support and register at C-support if they have long-term symptoms three months or longer after SARS-CoV-2 infection. This is in line with the clinical case definition of PCC established by the WHO [4]. From February 2022 onwards, patients registered at C-support long COVID were invited via email to complete an online questionnaire. The questionnaire was only available in the Dutch language. Data for this study were collected until September 2022.

Online informed consent was obtained from all participants for the usage of data in scientific research. The Medical Ethics Review Board of the Erasmus University Medical Centre approved the study protocol (MEC-2021-0751).

### Measures

#### Socio-demographic and medical characteristics

The questionnaire included the socio-demographic variables gender (male, female, unidentified, prefer not to say), age (in years), educational level (highest attained level of formal education), and ethnicity (Dutch, Turkish, Moroccan, Antillean, Surinamese, Indonesian, prefer not to say). Seven age categories were formed: 18–24 years, 25–34 years, 35–44 years, 45–54 years, 55–64 years, 65–74 years, and 75 years and older. Education level was categorised into three categories according to the International Standard Classification of Education (ISCED-97): low (ISCED 0, 1, and 2), middle (ISCED 3 and 4), and high (ISCED 5 and 6) [30]. Ethnicity was categorised as Dutch and other.

Additionally, the questionnaire included items on medical characteristics: self-reported pre-existing chronic conditions (e.g., asthma, chronic obstructive pulmonary disease (COPD), inflammatory bowel disease (IBD), consequences of a stroke, depression/anxiety, severe heart disease, arthrosis, rheumatism, severe back complaints, hypertension, cancer, diabetes, and thyroid abnormalities), height in centimetres, weight in kilogram, month of first SARS-CoV-2 infection, hospital admission for COVID-19, and vaccination status. Time since SARS-CoV-2 infection in months was calculated based on month of the first SARS-CoV-2 infection and date of completing the questionnaire. Body mass index (BMI) was derived from height and weight and consisted of the following categories: underweight (<18.5 kg/m^2^), normal weight (18.5–25 kg/m^2^), overweight (25–30 kg/m^2^), and obese (>30 kg/m^2^) [31]. Month of the first SARS-CoV-2 infection was used as a proxy for the dominant SARS-CoV-2 strain based on the registry of the Dutch National Institute for Public Health and the Environment [32].

#### Primary outcome measure

The questionnaire included a list of 34 health symptoms (Supplementary Material 1). The list was based on the literature (Supplementary Material 1) and checked by and supplemented with input from patients and healthcare professionals. For every health symptom, the participants were asked to indicate whether or not they were currently experiencing it or had previously experienced the specific symptom since their infection with COVID-19 by checking a box when the symptom was experienced.

#### Secondary outcome measures

Secondary outcome measures were the HRQoL and healthcare use. The HRQoL was measured with the EQ-5D-5L, a generic instrument consisting of five items: mobility, self-care, usual activities, pain/discomfort, and anxiety/depression [33]. For each item, there are five response categories: no problems, slight problems, moderate problems, severe problems, and extreme problems/unable to. Participants were asked to complete the EQ-5D-5L items for their health today (e.g., health status on the day of filling out the questionnaire). The EQ-5D utility score was calculated using a Dutch value set [34]. This score represents how good or bad someone’s health state is according to health state preferences of the general population. The utility score is anchored from 0 (a state as bad as being dead) to 1 (full health) [35]. The EQ-5D-5L also includes a standardised visual analogue scale: EQ-VAS. The EQ-VAS ranges from 0 (‘worst imaginable health’) to 100 (‘best imaginable health’) [33]. Participants were asked to score their health today on the EQ-VAS.

Additionally, the questionnaire included a list of 26 healthcare providers (supplementary material 2). The list was based on a list of healthcare providers previously used in research on Q-fever and checked and supplemented with input from C-support, patients, and healthcare professionals. Participants were asked to check a box for each healthcare provider they had a consultation with since the onset of COVID-19. Furthermore, participants were asked to indicate the number of consultations they had with each healthcare provider.

### Statistical analysis

Descriptive statistics were performed for socio-demographic and medical characteristics of the population. Frequencies and percentages were obtained for gender, age categories, education level, nationality, other chronic conditions, BMI, hospital admission, and vaccination status. Mean score, standard deviation, median, and interquartile range were obtained for age (continuous) and time since SARS-CoV-2 infection.

A principal component analysis (PCA) of the 34 reported PCC symptoms was performed to identify symptom groups. The Oblimin rotation method was used in the PCA. The number of principal components was fixed at 8 based on the results of a Monte Carlo stimulation that was conducted to determine the statistically correct number of principal components. Loading equal to or higher than 0.25 was considered significant. The principal components were saved as weighted average of the symptoms that loaded on each principal component.

The principal components were included in a K-means clustering algorithm. The clustering algorithm was used to find the optimal grouping of participants based on the principal components. By K-means clustering, each participant is assigned to the cluster or phenotype that has the nearest mean. K was set to 3 based on the iteration history and Bonferroni testing.

Descriptive statistics were performed for each phenotype. Frequencies and percentages were obtained for gender, age categories, education level, ethnicity, other chronic conditions, BMI, hospital admission, vaccination status, symptoms, and healthcare use. Mean score, standard deviation, median, and interquartile range were obtained for age (continuous), time since SARS-CoV-2 infection, number of symptoms, and number of healthcare providers. The median was reported because of non-normality of the data. The Kruskal–Wallis test was used to compare age (continuous), time since SARS-CoV-2 infection, EQ-VAS score, EQ-5D utility score, healthcare use, and number of symptoms among the three phenotypes. Chi-square tests were used to compare gender, age (categorical), education level, nationality, other chronic conditions, BMI, hospital admission, vaccination, and EQ-5D-5L dimension scores among the three phenotypes.

Data analysis was performed by IBM SPSS statistics version 28.0.1.0. A *p*-value of 0.05 was considered significant.

## Results

### Characteristics of participants

In total, 8630 of the 14,791 (58.3%) invited patients completed the questionnaire. Table 1 describes the characteristics of participants. The majority of participants was female (76.8%), and the median age was 48 (IQR = 16) years. Most participants received a high level of education (53.6%), followed by a middle level of education (34.5%) and a low level of education (11.7%). Over half of the participants did not have other chronic conditions (53.3%). Of all participants, 8.6% was admitted to the hospital; of those, the majority was not admitted to the intensive care unit (ICU). The vast majority of the participants was vaccinated against COVID-19 (92.2%), mostly after their initial SARS-CoV-2 infection. Most participants (60.1%) were first infected with SARS-CoV-2 before 15 February 2021, when no primary dominant strain was registered; therefore, the dominant strain is ‘other’ (Supplementary Table 1). Fewer participants were infected when Alpha (19.9%), Beta (11.0%), or Omicron (8.7%) was dominant.

**Table 1.** Socio-demographic and medical characteristics of participants (N = 8630)

Socio-demographic characteristics						
Characteristic		Total (n(%), n = 8630)	Phenotype 1 (n(%), n = 1116)	Phenotype 2 (n(%), n = 4437)	Phenotype 3 (n(%), n = 3077)	*p*-value
Gender	Male	1977 (22.9)	384 (34.4)	1147 (25.9)	446 (14.5)	<.001
	Female	6630 (76.8)	725 (65.0)	3279 (73.9)	2626 (85.3)	
	Other	14 (0.2)	5 (0.4)	7 (0.2)	2 (0.1)	
	Prefer not to say	9 (0.1)	2 (0.2)	4 (0.1)	3 (0.1)	
Age	Median (IQR)	48.0 (16)	51.5 (18)	49.0 (16)	47.0 (16)	
	Mean (SD)	47.4 (11.6)	49.9 (13.0)	47.9 (11.4)	45.9 (11.1)	<.001
Age (years)	18–24	215 (2.5)	31 (2.8)	101 (2.3)	83 (2.7)	<.001
	25–34	1168 (13.5)	144 (12.9)	548 (12.4)	476 (15.5)	
	35–44	1893 (21.9)	174 (15.6)	963 (21.7)	756 (24.6)	
	45–54	2770 (32.1)	321 (28.8)	1420 (32.0)	1029 (33.4)	
	55–64	2199 (25.5)	320 (28.7)	1224 (27.6)	655 (21.3)	
	65–74	337 (3.9)	102 (9.1)	165 (3.7)	70 (2.3)	
	75+	48 (0.6)	24 (2.2)	16 (0.4)	8 (0.3)	
Education level	Low	1012 (11.7)	151 (13.5)	515 (11.6)	346 (11.2)	<.001
	Middle	2977 (34.5)	304 (27.2)	1521 (34.3)	1152 (37.4)	
	High	4625 (53.6)	660 (59.1)	2392 (53.9)	1573 (51.1)	
	Unknown	16 (0.2)	1 (0.1)	9 (0.2)	6 (0.2)	
Ethnicity	Dutch	8300 (96.2)	1085 (97.2)	4293 (96.8)	2922 (95.0)	0.055
	Other	303 (3.5)	30 (2.7)	131 (2.9)	142 (4.6)	
	Prefer not to say	27 (0.3)	1 (0.1)	13 (0.3)	13 (0.4)	
Medical characteristics						
Other chronic conditions	No other chronic conditions	4603 (53.3)	655 (58.7)	2436 (54.9)	1512 (49.1)	<.001
	Other chronic conditions	4027 (46.7)	461 (41.3)	2001 (45.1)	1565 (50.9)	
Body mass index	Underweight (<18.5 kg/m^2^)	126 (1.5)	24 (2.2)	38 (0.9)	64 (2.1)	<.001
	Healthy weight (18.5–25 kg/m^2^)	3527 (40.9)	528 (47.3)	1751 (39.5)	1248 (40.6)	
	Overweight (25–30 kg/m^2^)	2917 (33.8)	360 (32.3)	1580 (35.6)	977 (31.8)	
	Obesity (>30 kg/m^2^)	2058 (23.8)	204 (18.3)	1067 (24.1)	787 (25.6)	
	Unknown	2 (0.0)				
Time since SARS-CoV-2 infection	Median (IQR)	14.0 (13)	14.0 (13)	14.0 (12)	15.0 (12)	
	Mean (SD)	14.46 (6.850)	14.36 (7.0)	14.01 (6.7)	15.14 (6.9)	<.001
Hospital admission	Yes	744 (8.6)	106 (9.5)	366 (8.2)	272 (8.8)	0.358
	No	7886 (91.4)	1010 (90.5)	4071 (91.8)	2805 (91.2)	
ICU admission	Yes	198 (2.3)	44 (3.9)	99 (2.2)	55 (1.8)	<.001
	No	546 (6.3)	62 (5.6)	267 (6.0)	217 (7.1)	
Vaccination	Yes	7958 (92.2)	1038 (93.0)	4167 (93.9)	2753 (89.5)	<.001
	No	592 (6.9)	66 (5.9)	237 (5.3)	289 (9.4)	
	Prefer not to say	80 (0.9)	12 (1.1)	33 (0.7)	35 (1.1)	

### Principal component analysis

The median number of symptoms reported by the total population is 18 (IQR = 9), with the top 3 most reported symptoms being fatigue, concentration problems, and decreased physical condition. Using principal component analysis, 8 patterns of the 34 patient-reported symptoms were identified (for loading data see Supplementary Table 2).

### Cluster analysis

**Figure 1.**
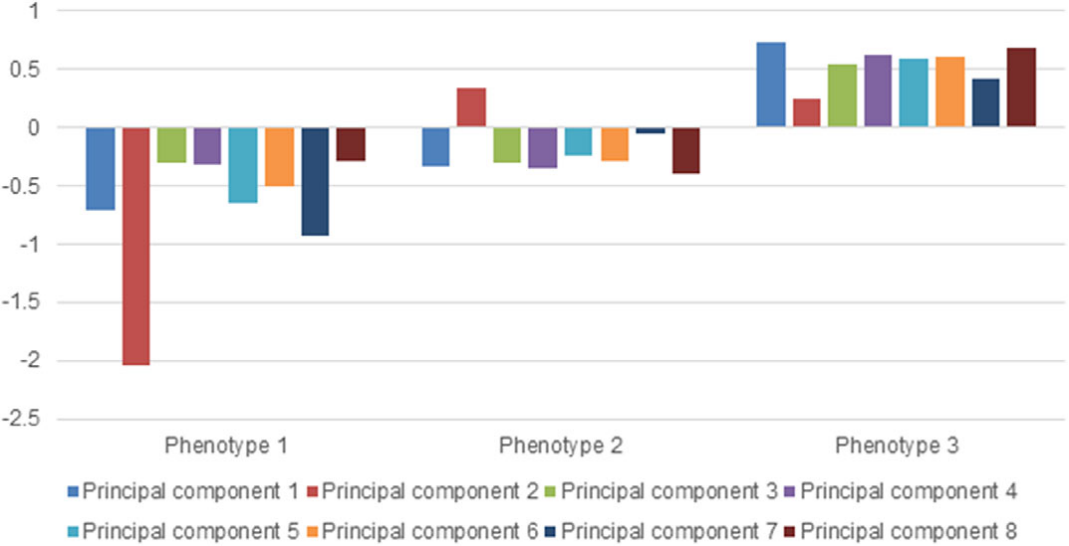
Principal component loading per phenotype.

Three phenotypes were identified. All participants were grouped into ‘phenotype 1’ (n = 1116), ‘phenotype 2’ (n = 4437), or ‘phenotype 3’ (n = 3077). Cluster allocation was based on the loading of each principal component. Phenotype 1 has participants with a lower-than-average number of symptoms allocated (Figure 1). Phenotype 2 has participants with an average number of symptoms allocated. Phenotype 3 has participants with a higher-than-average number of symptoms allocated.

The number of symptoms reported by participants significantly differed between phenotypes 1, 2, and 3 (*p* = 0.000) (Table 2). The highest median number of symptoms, 24 (IQR = 4), was reported in phenotype 3, while the lowest median number of symptoms, 9 (IQR = 5), was reported in phenotype 1. In phenotype 2, a median number of 16 symptoms was reported. The most frequently reported symptoms differ per phenotype (Supplementary Figure 1). The percentage of participants who reported each symptom is the highest for phenotype 3 on all reported symptoms.

**Table 2.** Number of reported symptoms by phenotype

	Cohort	Phenotype 1	Phenotype 2	Phenotype 3	*p*-value
Mean (SD)	17.71 (6.14)	9.32 (3.91)	15.36 (3.24)	24.14 (3.19)	<.001
Median (IQR)	18 (9)	9 (5)	16 (5)	24 (4)	
Minimum	0	0	6	16	
Maximum	33	19	22	33	

Compared to the other phenotypes, a significantly higher percentage of females was allocated to phenotype 3 (85.3%), and a significantly lower percentage of females was allocated to phenotype 1 (65.0%) (*p* < 0.001) (Table 1). Phenotype 1 has a significantly higher percentage of participants in age categories 65–74 (9.1%) and 75+ (2.2%) (*p* < 0.001). In phenotype 3, the percentage of participants who do not have other chronic conditions is significantly lower (49.1%) than that in phenotypes 1 and 2 (58.7 and 54.9%, respectively) (*p* < 0.001). The median duration of PCC was 14 months in phenotypes 1 and 2, and 15 months in phenotype 3; the difference between the phenotypes was significant (*p* < 0.001). The results showed no significant differences in the hospital admission rate between the phenotypes (9.5%, 8.2%, and 8.8%, respectively). In all phenotypes, the majority of participants was vaccinated against COVID-19; however, in phenotype 3, the percentage of participants who was vaccinated was significantly lower than that in the other phenotypes (*p* < 0.001). For each phenotype, most participants were infected before a single dominant strain was registered; therefore, ‘other’ is the dominant strain (62.4%, 57.2%, and 63.5%, respectively) (Supplementary Table 1). For each phenotype, this is followed by Alpha (16.4%, 21.8%, 18.4%), Beta (12.6%, 11.0%, 11.2%), and Omicron (8.7%, 10.0%, 6.9%), with the phenotypes differing significantly from each other (*p* < 0.001).

### Differences in HRQoL between phenotypes

**Figure 2.**
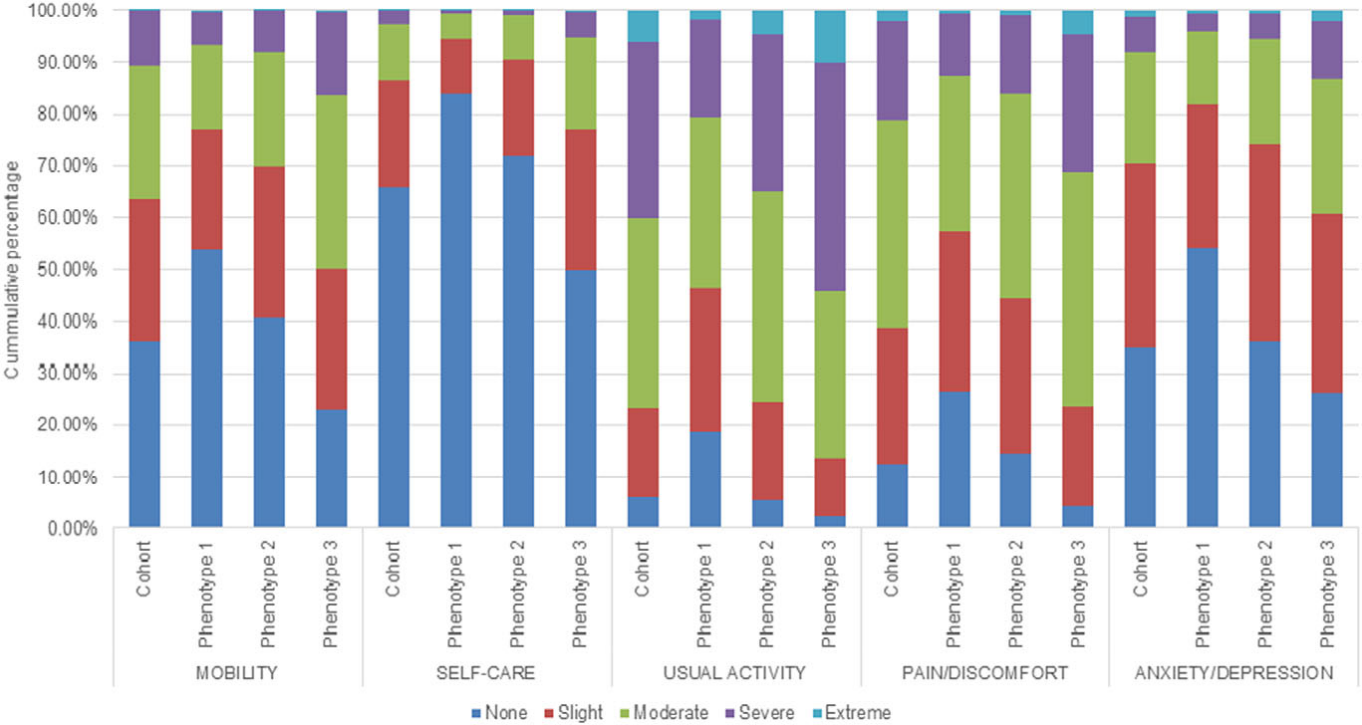
EQ-5D-5L dimensions by phenotype.

**Figure 3.**
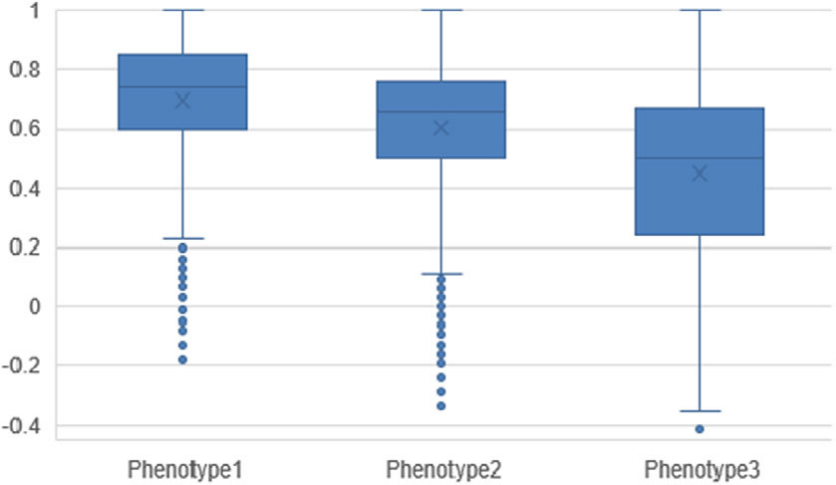
Descriptive statistics of the EQ-5D utility score by phenotype. X denotes mean, the line in the box denotes median, the box is the interquartile range, and the whiskers are the minimum and maximum points with outliers removed and depicted as dots.

**Figure 4.**
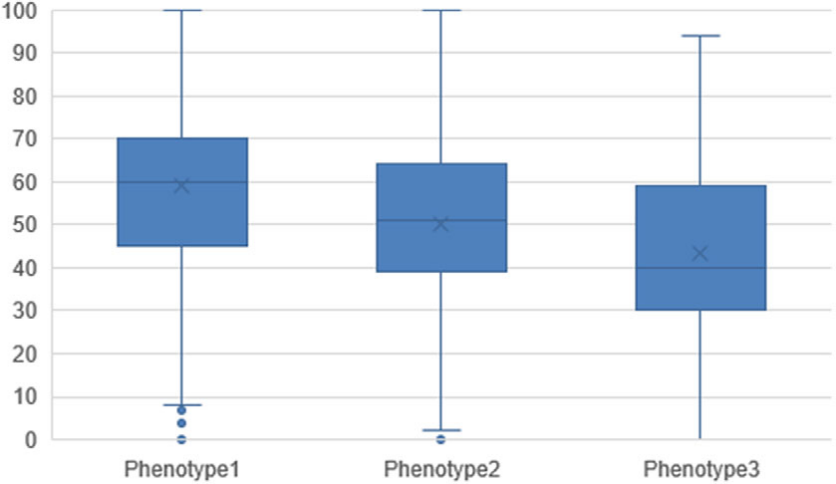
Descriptive statistics of the EQ-VAS score by phenotype. X denotes mean, the line in the box denotes median, the box is the interquartile range, and the whiskers are the minimum and maximum points with outliers removed and depicted as dots.

Figure 2 shows the distribution of the level of problems experienced on the EQ-5D-5L dimensions by phenotype. For each of the five dimensions, participants in phenotype 3 reported significantly more problems than participants in phenotypes 1 and 2 (Supplementary Table 3). Most problems were experienced for usual activities. The largest differences between phenotypes were seen for self-care between phenotype 1 and phenotype 3, with 28.0% and 50.1% of the participants experiencing no problems, respectively. The median EQ-5D utility (Figure 3) and EQ-VAS score (Figure 4) by phenotype differed significantly (Supplementary Table 4), with the lowest scores for participants classified into phenotype 3. The median EQ-5D utility score was 0.74 for phenotype 1, 0.66 for phenotype 2, and 0.50 for phenotype 3. The median EQ-VAS score was 60 for phenotype 1, 51 for phenotype 2, and 40 for phenotype 3.

### Differences in healthcare use between phenotypes

**Figure 5.**
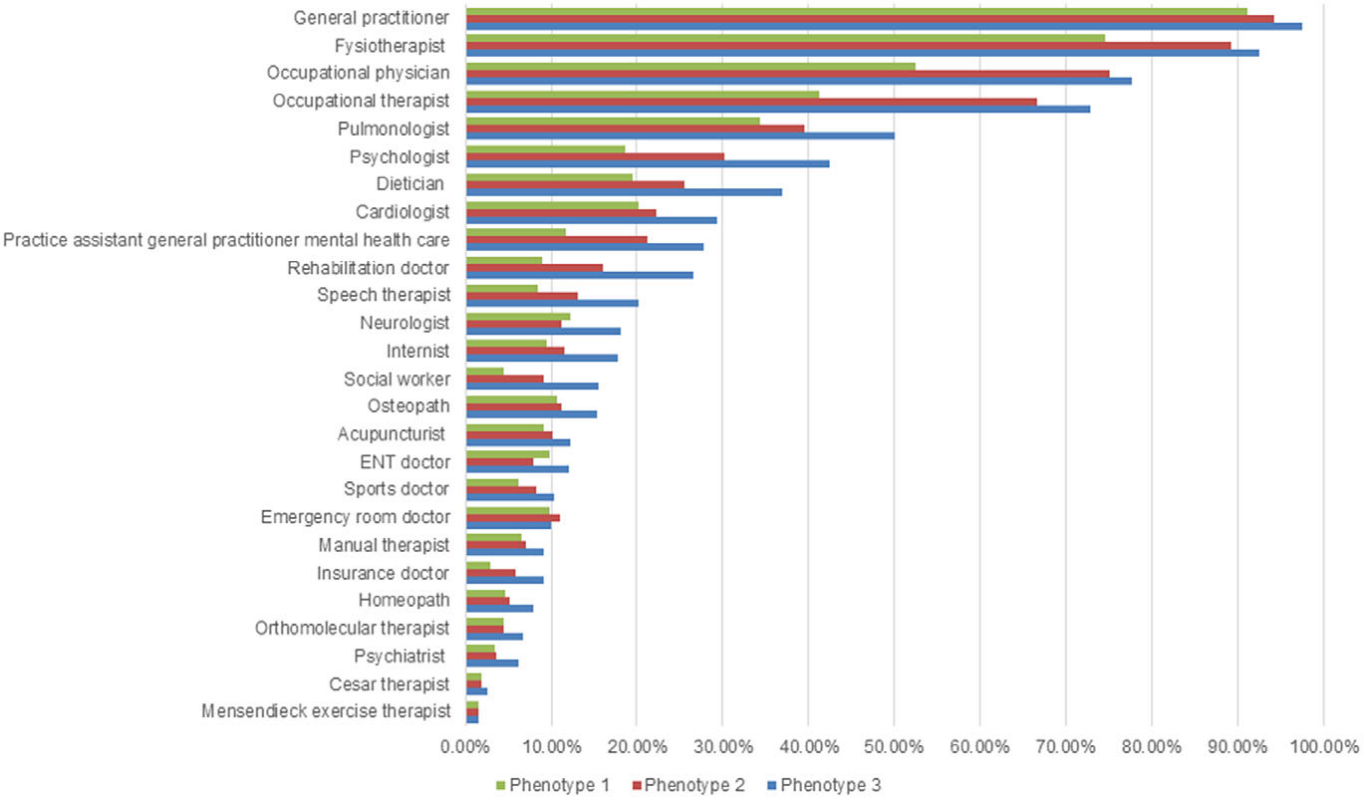
Percentage of participants who consulted each healthcare provider by phenotype.

The median number of different healthcare providers consulted by participants was the highest in phenotype 3, 7 (IQR = 4), and the lowest in phenotype 1, 4 (IQR = 3) (Supplementary Table 5). The percentage of participants who mentioned consulting any healthcare provider is the highest in phenotype 3 and the lowest in phenotype 1 for each of the listed healthcare providers (Figure 5). The average number of consultations per healthcare provider differs among the phenotypes (Supplementary Figure 2).

## Discussion

In this cross-sectional study of individuals with PCC, eight symptom patterns and three phenotypes were identified. These three phenotypes were driven by number of symptoms, rather than symptom pattern. As a result, each symptom pattern was present in each of the phenotypes identified. The main difference between the three phenotypes was the number of reported symptoms. In the phenotype with the highest number of symptoms, HRQoL was the poorest and healthcare use was the highest.

This finding is consistent with the results of a study by Bowyer et al. (2023) in which data from 9 longitudinal studies were used in a latent class analysis. Based on an extensive set of symptoms, they identified two patterns, one with a low prevalence of symptoms and the other with a higher prevalence of symptoms [27]. Other studies that aimed to identify symptom clusters of patterns yielded different results. Deep et al. (2021) found that clinical manifestation of PCC varies and has a wide range of symptoms. Yelin et al. (2022) found six symptom patterns based on the type of symptoms [26]. These symptom patterns are similar to the symptom patterns that we identified in our study through PCA. The current study used a principal component analysis followed by a K-means cluster analysis, yielding different final phenotypes. Furthermore, a Bayesian meta-regression of 54 studies and 2 medial record databases also found patterns based on the type of symptoms [3]. The Global Burden of Disease Long COVID Collaborators (2022) pre-defined clusters (persistent fatigue with bodily pain or mood swings, cognitive problems, or ongoing respiratory problems) and calculated the proportion of patients fitting in at least one of those clusters [36]. Another reason for the discrepancy in results may be the time between initial COVID-19 infection and data collection. The studies by Yelin et al. (2022) and the Global Burden of Disease Long COVID Collaborators (2022) reported a shorter time duration between infection and data collection than our study, with a median of 14 months. Furthermore, both studies used a shorter list of symptoms than the current study.

Additionally, this study compared socio-demographic and medical background characteristics, HRQoL, and healthcare use across the three phenotypes. The duration of PCC was slightly longer in the phenotype with a higher number of symptoms. This in line with the findings of the study by Bowyer et al. (2023), where the cluster with a higher burden appeared to be more common in individuals who had a COVID-19 infection more than 12 weeks before the study. There was no difference in the hospital admission rates between the phenotypes. The phenotype with the highest median number of symptoms had a higher percentage of female participants than the other two phenotypes and the study population, whereas in the phenotype with the lowest median number of symptoms, the percentage of males was higher than that in the study population. Bowyer et al. (2023) reported that the symptom pattern with a higher symptom burden is more common in females; this is coherent with our findings.

Results showed that participants in the phenotype with the highest number of symptoms have a lower vaccination rate than the other phenotypes. A study by Strain et al. (2022) showed a reduction of symptoms in patients with PCC after vaccination. The majority of the participants was vaccinated after their initial SARS-CoV-2 infection, while others were vaccinated before their initial SARS-CoV-2 infection. The vaccination rate in all three phenotypes is high compared to the vaccination rate in the Netherlands as a whole (82.1%) [37]. Even with a high vaccination rate in each of the phenotypes, it could be possible that the lower vaccination rate in this phenotype contributed to the high number of symptoms.

For the majority of participants in each of the phenotypes, no single dominant strain can be marked. When a dominant strain was found, Alpha was most found for each of the phenotypes. Results of a meta-analysis of PCC caused by different strains of SARS-CoV-2 including 51 studies show that the wild-type and different strains could all cause PCC [38]. In addition, generally, no differences in presentation of PCC were found between the different strains [38].

As expected, participants in the phenotype with the highest median number of symptoms experienced the worst HRQoL. The prevalence of problems on the EQ-5D-5L dimensions in the study population and the three phenotypes was higher than the prevalence of problems described in a meta-analysis of 12 studies on the HRQoL of PCC patients [20], indicating that the HRQoL is lower in the current study.

With the data from the current study, it is not known whether symptoms are solely caused by PCC or whether symptoms are, at least partly, caused by the indirect impact of the COVID pandemic, that is governmental measures against the spread of COVID-19, such as lockdown and quarantine measures, on health and well-being of the population. Wang et al. (2021) used a chain mediation model to show that the perceived impact of the COVID pandemic is a sequential mediator between symptoms and mental health outcome [39]. This could also be the case for the current study with HRQoL as the outcome.

Additionally, the mean score on the EQ-VAS in the study population and all three phenotypes was lower than the pooled EQ-VAS score reported in the meta-analysis [20]. These differences in findings may be due to a difference in the definition of PCC that was used. In this study, symptoms needed to be occurring for 3 months or longer after initial COVID-19 infection, whereas the study by Malik et al. did not include a restriction on the duration of symptoms. Moreover, the differences in findings may be due to differences in the study population. The studies included in the meta-analysis reported on participants who have been hospitalised with a COVID-19 infection, whereas only a small proportion of the participants in our study population has been hospitalised with COVID-19 infection, but they were registered in a long COVID-19 registry. The patients registered in the C-support long COVID-19 registry are generally patients with a healthcare demand. Compared to the results of a cross-sectional study among non-hospitalised PCC patients, the current study found similar HRQoL outcomes [40]. The phenotype with fewer complaints scored slightly higher on the EQ-VAS in our study than that in Meys et al. (2020), whereas the phenotypes with an average or a higher number of complaints scored slightly lower on the EQ-VAS. EQ-VAS scores for each of the phenotypes, as well as EQ-5D utility scores, were lower than the scores for the general population with COVID-19 [41]. Furthermore, when comparing EQ-VAS and EQ-5D utility scores of persons with post-COVID conditions to those of persons living with other medical conditions, it can be seen that each phenotype scores were lower on the EQ-VAS or EQ-5D utility in the study population than in patients suffering from diabetes [42], human immunodeficiency virus (HIV) [43], respiratory disease [44], dengue fever [45], and skin disease [46].

This study found that participants with PCC consulted between 0 and 21 healthcare providers, with a median of 6 healthcare providers. These results were in line with other studies advising a multi-disciplinary approach for PCC [24, 25]. A relatively high healthcare use was found in this study, which is supported by a study by Hedberg et al. (2022) which showed an increase in healthcare use by PCC patients in the 12 months after acute infection compared to that before COVID-19 infection [47]. Participants in the phenotype with fewer symptoms consulted fewer healthcare providers than those with more symptoms.

For future studies, it is recommended that a wide variety of symptoms experienced by PCC patients should be taken into account when investigating the long-term effects of COVID-19 and different phenotypic presentations of PCC should be further investigated. This could include taking into account differences in time since SARS-CoV-2 infection or gender. In addition, a clear definition of PCC is needed for coherent research into the causes, impact, and treatment of PCC. This study yet again highlights the complexity of PCC and the variety and amount of symptoms experienced by those suffering from PCC. Even though this study cannot provide a clear definition of what PCC entails, we hope the results of this study add to understanding PCC and its different phenotypes.

### Strengths and limitations

The most important strengths of this study include the large sample size of patients with PCC and the wide range of symptoms. Furthermore, using the combination of PCA and K-means clustering is a strength because both symptom patterns and PCC phenotypes were identified. Additionally, the link between symptom phenotypes and the outcome measures HRQoL and healthcare use adds to the existing literature on PCC clusters and subtypes. Insights into HRQoL and healthcare use of patients in different PCC phenotypes can help get a better understanding of PCC. The knowledge of PCC and different phenotypes or subgroups can be a basis for further research into healthcare and support needs of PCC patients. This could possibly help tailor care and support for patients with PCC, more to the specific needs of the phenotypes.

This study has several limitations. First, the study population might not be representative of the entire population of patients with PCC. The study population seems to underrepresent mostly or fully recovered patients, and patients with low numbers of PCC symptoms. PPC patients need to register at C-support themselves; therefore, mostly patients who are actively looking for help are included in this study. These are likely patients with most symptoms and also patients who are capable of finding C-support. Furthermore, although this study identified phenotypes based on the presence of symptoms, the severity of symptoms was not taken into account. A second limitation is the non-response bias and selection bias, which were possibly introduced in this study, especially in those with a longer time since COVID-19 infection. PCC patients who are mostly or fully recovered might be less inclined to fill out the questionnaire or they were not part of the C-support PCC registry. Furthermore, patients might be less likely to fill out the extensive questionnaire when they experience a lot of cognitive symptoms. This may have resulted in a very specific groups of PCC patients with severe symptoms having been included in this study. A non-response analysis was not performed because the data needed for this were not available; therefore, characteristics of patients who were invited to participate in this study but did not is not available. Another limitation is recall bias, especially in participants with a longer time between COVID-19 infection and data collection. It could be difficult to recollect all the symptoms they experienced over the entire period that they have been living with PCC, and therefore, it could be difficult to answer questions about the entire period of PCC. Furthermore, the way in which the questions about having symptoms were posed could pose a limitation. Respondents were asked which symptoms they had had since COVID-19 infection. Therefore, it is unknown whether all reported symptoms were present simultaneously and at the moment of filling out the questionnaire. Another limitation is not taking sex-specific symptoms into account. Menstrual complaints were included in the analyses of this study that included both men and women, even though menstrual complaints are a sex-specific symptom experienced by 15.7% of the total study population. Additionally, all patients were included in analysis, regardless of the time between SARS-CoV-2 infection and data collection. Therefore, it is unclear whether phenotype deferrers based on duration of PCC.

### Conclusion

In conclusion, patients with PCC experience a large variety of symptoms to a greater or lesser degree. Three specific phenotypes were identified based on symptom groups and differ in the number of symptoms patients experience. In the phenotype with participants who experience a higher number of symptoms, a lower HRQoL and higher healthcare use are found. This suggest there is a relationship between the number of symptoms and HRQoL and healthcare use. With these findings in mind, additional studies into specific healthcare needs and treatment strategies are needed to tailor treatment plans to patient’s needs.

## Supporting information

Gerritzen et al. supplementary material Gerritzen et al. supplementary material

## Data Availability

The dataset generated and analysed during the current study is available from the corresponding author on reasonable request.

## Supplementary material

For supplementary material accompanying this paper visit http://doi.org/10.1017/S0950268823001139. click here to view supplementary material

## References

[1] World Health Organization (2022) COVID-19 weekly epidemiological update, edition 122, 14 December 2022.

[2] Ballering AV, van Zon SKR, olde Hartman TC and Rosmalen JGM (2022). Persistence of somatic symptoms after COVID-19 in the Netherlands: An observational cohort study. Lancet 400(10350), 452–461. 35934007 10.1016/S0140-6736(22)01214-4 PMC9352274

[3] Global Burden of Disease Long CC (2022) Estimated global proportions of individuals with persistent fatigue, cognitive, and respiratory symptom clusters following symptomatic COVID-19 in 2020 and 2021. JAMA 328(16), 1604–1615. 36215063 10.1001/jama.2022.18931 PMC9552043

[4] World Health Organization (2021) A clinical case definition of post COVID-19 condition by a Delphi consensus, 6 October 2021. World Health Organization.

[5] Adler L, Gazit S, Pinto Y, Perez G, Mizrahi Reuveni M, Yehoshua I, Hoffman R, Azuri J and Patalon T (2022) Long-COVID in patients with a history of mild or asymptomatic SARS-CoV-2 infection: A Nationwide Cohort study. Scandinavian Journal of Primary Health Care 40(3), 342–349. 36314555 10.1080/02813432.2022.2139480 PMC9848375

[6] Doykov I, Hällqvist J, Gilmour KC, Grandjean L, Mills K and Heywood WE (2020) ‘The long tail of Covid-19’ - The detection of a prolonged inflammatory response after a SARS-CoV-2 infection in asymptomatic and mildly affected patients. F1000Research 9, 1349. 33391730 10.12688/f1000research.27287.1 PMC7745182

[7] Faghy MA, Owen R, Thomas C, Yates J, Ferraro FV, Skipper L, Barley-McMullen S, Brown DA, Arena R and Ashton REM (2022) Is long COVID the next global health crisis? Journal of Global Health 12, 03067. 36285549 10.7189/jogh.12.03067 PMC9597397

[8] Wulf Hanson S, Abbafati C, Aerts JG, Al-Aly Z, Ashbaugh C, Ballouz T, Blyuss O, Bobkova P, Bonsel G, Borzakova S, Buonsenso D, Butnaru D, Carter A, Chu H, De Rose C, Diab MM, Ekbom E, El Tantawi M, Fomin V, Frithiof R, Gamirova A, Glybochko PV, Haagsma JA, Javanmard SH, Hamilton EB, Harris G, Heijenbrok-Kal MH, Helbok R, Hellemons ME, Hillus D, Huijts SM, Hultström M, Jassat W, Kurth F, Larsson I-M, Lipcsey M, Liu C, Loflin CD, Malinovschi A, Mao W, Mazankova L, McCulloch D, Menges D, Mohammadifard N, Munblit D, Nekliudov NA, Ogbuoji O, Osmanov IM, Peñalvo JL, Petersen MS, Puhan MA, Rahman M, Rass V, Reinig N, Ribbers GM, Ricchiuto A, Rubertsson S, Samitova E, Sarrafzadegan N, Shikhaleva A, Simpson KE, Sinatti D, Soriano JB, Spiridonova E, Steinbeis F, Svistunov AA, Valentini P, van de Water BJ, van den Berg-Emons R, Wallin E, Witzenrath M, Wu Y, Xu H, Zoller T, Adolph C, Albright J, Amlag JO, Aravkin AY, Bang-Jensen BL, Bisignano C, Castellano R, Castro E, Chakrabarti S, Collins JK, Dai X, Daoud F, Dapper C, Deen A, Duncan BB, Erickson M, Ewald SB, Ferrari AJ, Flaxman AD, Fullman N, Gamkrelidze A, Giles JR, Guo G, Hay SI, He J, Helak M, Hulland EN, Kereselidze M, Krohn KJ, Lazzar-Atwood A, Lindstrom A, Lozano R, Magistro B, Malta DC, Månsson J, Mantilla Herrera AM, Mokdad AH, Monasta L, Nomura S, Pasovic M, Pigott DM, Reiner Jr RC, Reinke G, Ribeiro ALP, Santomauro DF, Sholokhov A, Spurlock EE, Walcott R, Walker A, Wiysonge CS, Zheng P, Bettger JP, Murray CJ and Vos T (2022) A global systematic analysis of the occurrence, severity, and recovery pattern of long COVID in 2020 and 2021. medRxiv.

[9] Ani N, Amar DD and Elaine YW (2023) Post-COVID-19 condition. Annual Review of Medicine 74(1), 55–64. 10.1146/annurev-med-043021-030635 35914765

[10] Deer RR, Rock MA, Vasilevsky N, Carmody L, Rando H, Anzalone AJ, Basson MD, Bennett TD, Bergquist T, Boudreau EA, Bramante CT, Byrd JB, Callahan TJ, Chan LE, Chu H, Chute CG, Coleman BD, Davis HE, Gagnier J, Greene CS, Hillegass WB, Kavuluru R, Kimble WD, Koraishy FM, Köhler S, Liang C, Liu F, Liu H, Madhira V, Madlock-Brown CR, Matentzoglu N, Mazzotti DR, McMurry JA, McNair DS, Moffitt RA, Teshamae S, Monteith RA, Parker AM, Perry MA, Pfaff E, Reese JT, Saltz J, Schuff RA, Solomonides AE, Solway J, Spratt H, Stein GS, Sule AA, Topaloglu U, Vavougios GD, Wang L, Haendel MA and Robinson PN (2021) Characterizing long COVID: Deep phenotype of a complex condition. eBioMedicine 74, 103722. 34839263 10.1016/j.ebiom.2021.103722 PMC8613500

[11] Nasserie T, Hittle M and Goodman SN (2021) Assessment of the frequency and variety of persistent symptoms among patients with COVID-19: A systematic review. JAMA Network Open 4(5), e2111417. 34037731 10.1001/jamanetworkopen.2021.11417 PMC8155823

[12] Salamanna F, Veronesi F, Martini L, Landini MP and Fini M (2021) Post-COVID-19 syndrome: The persistent symptoms at the post-viral stage of the disease. A systematic review of the current data. Frontiers in Medicine 8, 653516. 34017846 10.3389/fmed.2021.653516 PMC8129035

[13] del Rio C, Collins LF and Malani P (2020) Long-term health consequences of COVID-19. JAMA 324(17), 1723–1724. 33031513 10.1001/jama.2020.19719 PMC8019677

[14] Nehme M, Braillard O, Chappuis F, Courvoisier DS and Guessous I (2021) Prevalence of symptoms more than seven months after diagnosis of symptomatic COVID-19 in an outpatient setting. Annals of Internal Medicine 174(9), 1252–1260. 34224254 10.7326/M21-0878 PMC8280535

[15] Davis HE, McCorkell L, Vogel JM and Topol EJ (2023). Long COVID: Major findings, mechanisms and recommendations. Nature Reviews Microbiology 21(3), 133–146. 36639608 10.1038/s41579-022-00846-2 PMC9839201

[16] Han Q, Zheng B, Daines L and Sheikh A (2022) Long-term sequelae of COVID-19: A systematic review and meta-analysis of one-year follow-up studies on post-COVID symptoms. Pathogens 11(2), 269. 35215212 10.3390/pathogens11020269 PMC8875269

[17] Taquet M, Geddes JR, Husain M, Luciano S and Harrison PJ (2021) 6-month neurological and psychiatric outcomes in 236 379 survivors of COVID-19: A retrospective cohort study using electronic health records. Lancet Psychiatry 8(5), 416–427. 33836148 10.1016/S2215-0366(21)00084-5 PMC8023694

[18] Penninx BWJH, Benros ME, Klein RS and Vinkers CH (2022) How COVID-19 shaped mental health: From infection to pandemic effects. Nature Medicine 28(10), 2027–2037. 10.1038/s41591-022-02028-2 PMC9711928 36192553

[19] Crook H, Raza S, Nowell J, Young M and Edison P (2021) Long COVID—Mechanisms, risk factors, and management. BMJ 374, 1648. 10.1136/bmj.n1648 34312178

[20] Malik P, Patel K, Pinto C, Jaiswal R, Tirupathi R, Pillai S and Patel U (2022) Post-acute COVID-19 syndrome (PCS) and health-related quality of life (HRQoL): A systematic review and meta-analysis. Journal of Medical Virology 94(1), 253–262. 34463956 10.1002/jmv.27309 PMC8662132

[21] Fischer A, Zhang L, Elbéji A, Wilmes P, Oustric P, Staub T, Nazarov PV, Ollert M and Fagherazzi G (2022) Long COVID symptomatology after 12 months and its impact on quality of life according to initial coronavirus disease 2019 disease severity. Open Forum Infectious Diseases 9(8), ofac397. 35983269 10.1093/ofid/ofac397 PMC9379809

[22] Menges D, Ballouz T, Anagnostopoulos A, Aschmann HE, Domenghino A, Fehr JS and Puhan MA (2021) Burden of post-COVID-19 syndrome and implications for healthcare service planning: A population-based cohort study. PLoS One 16(7), e0254523. 34252157 10.1371/journal.pone.0254523 PMC8274847

[23] McNaughton CD, Austin PC, Sivaswamy A, Fang J, Abdel-Qadir H, Daneman N, Udell JA, Wodchis WP, Mostarac I, Lee DS and Atzema CL (2022) Post-acute health care burden after SARS-CoV-2 infection: A retrospective cohort study. Canadian Medical Association Journal 194(40), E1368–E1376. 36252983 10.1503/cmaj.220728 PMC9616149

[24] Raveendran AV, Jayadevan R and Sashidharan S (2021) Long COVID: An overview. Diabetes & Metabolic Syndrome: Clinical Research & Reviews 15(3), 869–875. 10.1016/j.dsx.2021.04.007 PMC8056514 33892403

[25] Greenhalgh T, Knight M, A’Court C, Buxton M and Husain L (2020) Management of post-acute COVID-19 in primary care. BMJ 370, m3026. 32784198 10.1136/bmj.m3026

[26] Yelin D, Margalit I, Nehme M, Bordas-Martínez J, Pistelli F, Yahav D, Guessous I, Durà-Miralles X, Carrozzi L, Shapira-Lichter I, Vetter P, Peleato-Catalan D, Tiseo G, Wirtheim E, Kaiser L, Gudiol C, Falcone M, Leibovici L and on behalf of the LongCOV Research Group (2022) Patterns of long COVID symptoms: A multi-center cross sectional study. Journal of Clinical Medicine 11(4), 898. 35207171 10.3390/jcm11040898 PMC8875229

[27] Bowyer RCE, Huggins C, Toms R, Shaw RJ, Hou B, Thompson EJ, Kwong ASF, Williams DM, Kibble M, Ploubidis GB, Timpson NJ, Sterne JAC, Chaturvedi N, Steves CJ, Tilling K, Silverwood RJ (2023) CONVALESCENCE Study. Characterising patterns of COVID-19 and long COVID symptoms: evidence from nine UK longitudinal studies. Eur J Epidemiol. Feb;38(2), 199–210. doi: 10.1007/s10654-022-00962-6. Epub 2023 Jan 21. PMID: 36680646; PMCID: PMC9860244. 10.1007/s10654-022-00962-6 36680646 PMC9860244

[28] Kenny G, McCann K, O’Brien C, Savinelli S, Tinago W, Yousif O, Lambert JS, O’Broin C, Feeney ER, De Barra E, Doran P, Mallon PWG and All-Ireland Infectious Diseases (AIID) Cohort Study Group (2022) Identification of distinct long COVID clinical phenotypes through cluster analysis of self-reported symptoms. Open Forum Infectious Diseases 9(4), ofac060. 35265728 10.1093/ofid/ofac060 PMC8900926

[29] Whitaker M, Elliott J, Chadeau-Hyam M, Riley S, Darzi A, Cooke G, Ward H and Elliott P (2022) Persistent COVID-19 symptoms in a community study of 606,434 people in England. Nature Communications 13(1), 1957. 10.1038/s41467-022-29521-z PMC9005552 35413949

[30] CBS (2021) Standaard onderwijsindeling 2021. CBS.

[31] Obesity WHOCo, World Health Organization (2000) Obesity: Preventing and Managing the Global Epidemic: Report of a WHO Consultation. Geneva: World Health Organization. 11234459

[32] Milieu RvVe (2023) Varianten van het coronavirus SARS-CoV-2: Rijksinstituut voor Volksgezondheid en Milieu [updated 21 March 2023]. Available from: https://www.rivm.nl/coronavirus-covid-19/virus/varianten

[33] Herdman M, Gudex C, Lloyd A, Janssen MF, Kind P, Parkin D, Parkin D, Bonsel G and Badia X (2011) Development and preliminary testing of the new five-level version of EQ-5D (EQ-5D-5L). Quality of Life Research 20(10), 1727–1736. 21479777 10.1007/s11136-011-9903-x PMC3220807

[34] Versteegh MM, Vermeulen KM, Evers SMAA, de Wit GA, Prenger R and Stolk EA (2016) Dutch tariff for the five-level version of EQ-5D. Value in Health 19(4), 343–352. 27325326 10.1016/j.jval.2016.01.003

[35] Foundation ER (2019) EQ-5D-5L User Guide. EuroQol Research Foundation. https://euroqol.org/publications/user-guides/

[36] Strain WD, Sherwood O, Banerjee A, Van der Togt V, Hishmeh L and Rossman J (2022) The impact of COVID vaccination on symptoms of long COVID: An international survey of people with lived experience of long COVID. Vaccines 10(5), 652. 35632408 10.3390/vaccines10050652 PMC9146071

[37] RIVM RvVeM (2023) Deelname COVID-19-vaccinatie in Nederland.

[38] Du M, Ma Y, Deng J, Liu M and Liu J (2022) Comparison of long COVID-19 caused by different SARS-CoV-2 strains: A systematic review and meta-analysis. International Journal of Environmental Research and Public Health 19(23), 16010. 36498103 10.3390/ijerph192316010 PMC9736973

[39] Wang C, Chudzicka-Czupała A, Tee ML, Núñez MIL, Tripp C, Fardin MA, Habib HA, Tran BX, Adamus K, Anlacan J, Aparicio García ME, Grabowski D, Hussain S, Hoang MT, Hetnał M, Le XT, Ma W, Pham HQ, Reyes PWC, Shirazi M, Tan Y, Tee CA, Xu L, Xu Z, Vu GT, Zhou D, Chan NA, Kuruchittham V, McIntyre RS, Ho CSH, Ho R and Sears SF (2021) A chain mediation model on COVID-19 symptoms and mental health outcomes in Americans, Asians and Europeans. Scientific Reports 11(1), 6481. 33742072 10.1038/s41598-021-85943-7 PMC7979938

[40] Meys R, Delbressine JM, Goërtz YMJ, Vaes AW, Machado FVC, Van Herck M, Burtin C, Posthuma R, Spaetgens B, Franssen FME, Spies Y, Vijlbrief H, Van’t Hul AJ, Janssen DJA, Spruit MA and Houben-Wilke S (2020) Generic and respiratory-specific quality of life in non-hospitalized patients with COVID-19. Journal of Clinical Medicine 9(12), 3993. 33317214 10.3390/jcm9123993 PMC7764406

[41] Tran BX, Nguyen HT, Le HT, Latkin CA, Pham HQ, Vu LG, Le X.T.T, Nguyen TT, Pham QT, Ta N.T.K, Nguyen QT, Ho C.S.H and Ho R.C.M (2020) Impact of COVID-19 on economic well-being and quality of life of the Vietnamese during the national social distancing. Frontiers Psychology 11, 565153. 10.3389/fpsyg.2020.565153 PMC7518066 33041928

[42] Nguyen HTT, Moir MPI, Nguyen TX, Vu AP, Luong LH, Nguyen TN, Nguyen LH, Tran BX, Tran TT, Latkin CA, Zhang MWB, Ho RCM and Vu HTT (2018) Health-related quality of life in elderly diabetic outpatients in Vietnam. Patient Preference and Adherence 12, 1347–1354. 30100711 10.2147/PPA.S162892 PMC6067618

[43] Tran BX, Dang AK, Truong NT, Ha GH, Nguyen HLT, Do HN, Nguyen TQ, Latkin CA, Ho C.S.H and Ho R.C.M (2018) Depression and quality of life among patients living with HIV/AIDS in the era of universal treatment access in Vietnam. International Journal of Environmental Research and Public Health 15(12), 2888. 30562949 10.3390/ijerph15122888 PMC6313339

[44] Ngo CQ, Phan PT, Vu GV, Pham QLT, Nguyen LH, Vu GT, Tran TT, Nguyen HLT, Tran BX, Latkin CA, Ho CSH and Ho RCM (2019) Effects of different comorbidities on health-related quality of life among respiratory patients in Vietnam. Journal of Clinical Medicine 8(2), 214. 30736474 10.3390/jcm8020214 PMC6406871

[45] Tran BX, Thu Vu G, Hoang Nguyen L, Tuan Le Nguyen A, Thanh Tran T, Thanh Nguyen B, Thi Thai TP, Latkin CA, Ho CSH and Ho RCM (2018) Cost-of-illness and the health-related quality of life of patients in the dengue fever outbreak in Hanoi in 2017. International Journal of Environmental Research and Public Health 15(6), 1174. 29874790 10.3390/ijerph15061174 PMC6025163

[46] Nguyen SH, Nguyen LH, Vu GT, Nguyen CT, Le THT, Tran BX, Latkin CA, Ho CSH and Ho RCM (2019) Health-related quality of life impairment among patients with different skin diseases in Vietnam: A cross-sectional study. International Journal of Environmental Research and Public Health 16(3), 305. 30678097 10.3390/ijerph16030305 PMC6388287

[47] Hedberg P, Granath F, Bruchfeld J, Askling J, Sjöholm D, Fored M, Färnert A and Naucler P (2023) Post COVID-19 condition diagnosis: A population-based cohort study of occurrence, associated factors, and healthcare use by severity of acute infection. Journal of Internal Medicine 293(2), 246–258. 36478477 10.1111/joim.13584 PMC9877994

