# Supplementary material for: Identification of post-COVID-19 condition phenotypes, and differences in health-related quality of life and healthcare use: a cluster analysis
Source: Epidemiol Infect. 2023 Jul 18;151:e123. doi: 10.1017/S0950268823001139 (PMC10540165; doi:10.1017/S0950268823001139)
Supplement: Gerritzen et al. supplementary material [file S0950268823001139sup001.docx]

**Supplementary materials**

**Identification of post COVID-19 condition phenotypes, and differences in health-related quality of life and health care use: a cluster analysis**

**Authors: Iris Gerritzen^1*^, Iris M. Brus^1^, Inge Spronk^1^ Sara Biere-Rafi^2^, Suzanne Polinder^1^, and Juanita A. Haagsma^1^**

^1^ Department of Public Health, Erasmus University Medical Center, Rotterdam, the Netherlands

^2^ C-support, ‘s Hertogenbosch, the Netherlands

**^*^ Correspondence:**

Iris Gerritzen

i.gerritzen@erasmusmc.nl

Supplementary materials 1 – List of health symptoms and literature

| - Anxiety - Chest pain - Concentration problems - Confusion - Coughing - Coughing up mucus - Decreased physical condition - Depression - Diarrhoea - Difficulty with stimulus processing - Dizziness - Fatigue - Fever - Hair loss - Headache - Irritability - Joint pain | - Loss of appetite - Loss of smell - Loss of taste - Memory problems - Muscle ache - Muscle weakness - Nausea - Pain in limbs (arms, legs, hands, and feet) - Palpitations - Period complaints - Shortness of breath on exertion - Shortness of breath on rest - Skin conditions - Sleep problems - Sore throath - Tingeling in limbs (arms, legs, hands, and feet) - Trouble finding words |
| --- | --- |

*Literature base for symptoms:*

C-Support. C-Support Managementrapportage juli 2021.

Cabrera Martimbianco, A. L., Pacheco, R. L., Bagattini Â, M., & Riera, R. (2021). Frequency, signs and symptoms, and criteria adopted for long COVID-19: A systematic review. Int J Clin Pract, e14357.

Crook, H., Raza, S., Nowell, J., Young, M., & Edison, P. (2021). Long covid-mechanisms, risk factors, and management. Bmj, 374, n1648.

GBD, L. C. C. (2021). Surviving COVID-19: a global systematic analysis of long COVID disability in 2020.

Iqbal, F. M., Lam, K., Sounderajah, V., Clarke, J. M., Ashrafian, H., & Darzi, A. (2021). Characteristics and predictors of acute and chronic post-COVID syndrome: A systematic review and meta-analysis. EClinicalMedicine, 36, 100899.

Lopez-Leon, S., Wegman-Ostrosky, T., Perelman, C., Sepulveda, R., Rebolledo, P. A., Cuapio, A., & Villapol, S. (2021). More than 50 long-term effects of COVID-19: a systematic review and meta-analysis. Sci Rep, 11(1), 16144.

Nalbandian, A., Sehgal, K., Gupta, A., Madhavan, M. V., McGroder, C., Stevens, J. S., . . . Wan, E. Y. (2021). Post-acute COVID-19 syndrome. Nat Med, 27(4), 601-615.

Pavli, A., Theodoridou, M., & Maltezou, H. C. (2021). Post-COVID syndrome: Incidence, clinical spectrum, and challenges for primary healthcare professionals. Arch Med Res, 52(6), 575-581.

Salamanna, F., Veronesi, F., Martini, L., Landini, M. P., & Fini, M. (2021). Post-COVID-19 Syndrome: The Persistent Symptoms at the Post-viral Stage of the Disease. A Systematic Review of the Current Data. Front Med (Lausanne), 8, 653516.

van Kessel, S. A. M., Olde Hartman, T. C., Lucassen, P., & van Jaarsveld, C. H. M. (2021). Post-acute and long-COVID-19 symptoms in patients with mild diseases: a systematic review. Fam Pract.

Supplementary materials 2 – list of healthcare providers

| - Acupuncturist - Cardiologist - Cesar therapist - Dietician - Emergency room doctor - ENT doctor - Physiotherapist - General practitioner - Homeopath - Insurance doctor - Internist - Manual therapist - Mensendieck exercise therapist - Neurologist | - Occupational physician - Occupational therapist - Orthomolecular therapist - Osteopath - Practice assistant general practitioner mental health care - Psychiatrist - Psychologist - Pulmonologist - Rehabilitation doctor - Social worker - Speech therapist - Sports doctor |
| --- | --- |

**Supplementary table 1.** Principal component loadings

|  | **Component** | | | | | | | |
| --- | --- | --- | --- | --- | --- | --- | --- | --- |
|  | **1** | **2** | **3** | **4** | **5** | **6** | **7** | **8** |
| **Pain in limbs (arms, legs, hands, and feet)** | .866 | -.007 | -.003 | -.014 | -.060 | .002 | -.037 | -.031 |
| **Muscle ache** | .816 | -.004 | .016 | .037 | -.063 | -.069 | -.039 | -.117 |
| **Joint pain** | .802 | .006 | .011 | -.020 | -.014 | .024 | -.047 | -.026 |
| **Tingling in limbs** | .500 | -.076 | .005 | -.034 | .093 | -.032 | .057 | .155 |
| **Muscle weakness** | .448 | .047 | .024 | .010 | .093 | .081 | .203 | .080 |
| **Concentration problems** | -.006 | .766 | .008 | -.009 | .008 | -.033 | -.017 | .024 |
| **Memory problems** | .010 | .753 | .051 | .046 | .018 | .060 | -.055 | .095 |
| **Trouble finding words** | .046 | .618 | .025 | .054 | .030 | .021 | -.028 | .171 |
| **Difficulty with stimulus processing** | -.049 | .568 | .006 | -.069 | .116 | -.229 | .052 | -.041 |
| **Fatigue** | .034 | .361 | -.043 | -.003 | -.040 | -.074 | .325 | -.126 |
| **Loss of taste** | .005 | .018 | .953 | -.033 | -.029 | .057 | -.008 | -.053 |
| **Loss of smell** | -.007 | .025 | .952 | -.045 | -.043 | .070 | -.013 | -.054 |
| **Loss of appetite** | .030 | -.077 | .358 | .092 | .161 | -.119 | .006 | .170 |
| **Coughing** | -.020 | .029 | -.004 | .731 | -.023 | .021 | .128 | .037 |
| **Coughing up mucus** | .039 | -.039 | -.024 | .711 | .127 | .157 | -.023 | .043 |
| **Sore throat** | .030 | .032 | .065 | .550 | -.061 | -.343 | .052 | -.066 |
| **Fever** | .034 | .055 | .132 | .437 | -.077 | -.314 | .039 | -.003 |
| **Depression** | -.057 | .026 | .000 | .027 | .834 | .060 | -.019 | -.139 |
| **Anxiety** | .007 | -.129 | .000 | -.013 | .781 | .032 | .048 | .005 |
| **Irritability** | .101 | .343 | .009 | -.016 | .409 | -.136 | -.034 | -.082 |
| **Confusion** | .038 | .216 | .048 | .060 | .409 | -.009 | -.038 | .092 |
| **Sleep problems** | .079 | .120 | .023 | -.031 | .270 | -.085 | .132 | .103 |
| **Headache** | .072 | .204 | .012 | .060 | -.037 | -.621 | -.011 | -.108 |
| **Dizziness** | .090 | .052 | -.005 | -.011 | .089 | -.506 | .082 | .095 |
| **Nausea** | .104 | -.071 | .058 | .083 | .083 | -.499 | -.013 | .188 |
| **Shortness of breath on exertion** | .032 | .101 | .015 | .104 | -.024 | .193 | .672 | -.004 |
| **Chest pain** | .082 | -.157 | .028 | .031 | .029 | -.235 | .557 | .066 |
| **Shortness of breath on rest** | .003 | -.094 | .036 | .249 | .127 | .018 | .546 | -.013 |
| **Palpitations** | .037 | -.087 | .030 | -.181 | .082 | -.314 | .500 | .160 |
| **Decreased physical condition** | .076 | .334 | -.003 | .020 | -.036 | .138 | .452 | -.007 |
| **Hair loss** | -.012 | .089 | .062 | .036 | -.079 | .135 | .048 | .701 |
| **Skin conditions** | .162 | .051 | -.034 | .091 | .022 | .080 | -.036 | .562 |
| **Period complaints** | -.067 | .034 | .017 | -.089 | -.011 | -.184 | .039 | .505 |
| **Diarrhoea** | .068 | -.027 | -.047 | .227 | .078 | -.237 | -.080 | .304 |

The first principal component was a musculoskeletal pattern with symptoms of pain in the limbs, tingling of the limbs, joint pain, and muscle ache. The second was a cognitive pattern with concentration problems, memory problems, trouble finding words, difficulty with stimulus processing, and fatigue. The third included loss of smell, loss of taste, and loss of appetite. The fourth was a respiratory pattern of coughing, coughing up mucus, sore throat, and fever. Fifth was a mental pattern of depression, anxiety, irritability, confusion, and sleep problems. The sixth included headache, dizziness, and nausea. The seventh was a cardio-pulmonary pattern of shortness of breath on exertion, shortness of breath on rest, decreased physical condition, chest pain, and palpitations. The eighth and final principal component included hair loss, skin condition, period problems, and diarrhoea.

**Supplementary table 2.** Dominant SARS-CoV-2 strains per cluster

|  | Total (n(%), N=8630) | Phenotype 1 (n(%), n = 1116) | Phenotype 2 (n(%), n = 4437) | Phenotype 3 (n(%), n = 3077) | p-value |
| --- | --- | --- | --- | --- | --- |
| Other (until 15 February 2021) | 5187 (60.1) | 694 (62.4) | 2538 (57.2) | 1955 (63.5) | <.001 |
| Alpha variant (15 February 2021 – 27 July 2021) | 1717 (19.9) | 182 (16.4) | 968 (21.8) | 567 (18.4) |  |
| Beta variant (28 July 2021 – 26 December 2021) | 970 (11.2) | 140 (12.6) | 486 (11.0) | 344 (11.2) |  |
| Omikron variant (from 27 December 2021) | 750 (8.7) | 97 (8.7) | 442 (10.0) | 211 (6.9) |  |

**Supplementary figure 1.** Percentage of complaints by phenotype

**Supplementary table 3.** Descriptive statistics number of health care providers

|  | Cohort | Phenotype 1 | Phenotype 2 | Phenotype 3 | P-value | |
| --- | --- | --- | --- | --- | --- | --- |
| Median (IQR) | 6 (4) | 4 (3) | 6 (4) | 7 (4) |  | |
| Mean (SD) | 6.34 (3.0) | 4.79 (2.7) | 6.03 (2.7) | 7.37 (3.1) |  | <.001 |
| Minimum | 0 | 0 | 0 | 0 |  |  |
| Maximum | 21 | 16 | 17 | 21 |  |  |

**Supplementary figure 2.** Number of consultations per healthcare provider by phenotype

**Supplementary table 4.** Descriptive statistics EQ-5D-5L dimensions by phenotype

|  | | Total (n(%), N=8630) | Phenotype 1 (n(%), n = 1116) | Phenotype 2 (n(%), n = 4437) | Phenotype 3 (n(%), n = 3077) | p-value |
| --- | --- | --- | --- | --- | --- | --- |
| Mobility | None | 3121 (36.2) | 603 (54.0) | 1813 (40.9) | 705 (22.9) | <.001 |
|  | Slight | 2387 (27.7) | 260 (23.3) | 1285 (29.0) | 842 (27.4) |  |
|  | Moderate | 2183 (25.3) | 179 (16.0) | 986 (22.2) | 1018 (33.1) |  |
|  | Severe | 919 (10.6) | 71 (6.4) | 348 (7.8) | 500 (16.2) |  |
|  | Extreme | 20 (0.2) | 3 (0.3) | 5 (0.1) | 12 (0.4) |  |
| Self-care | None | 5666 (65.7) | 936 (83.9) | 3194 (72.0) | 1536 (49.9) | <.001 |
|  | Slight | 1789 (20.7) | 119 (10.7) | 831 (18.7) | 839 (27.3) |  |
|  | Moderate | 959 (11.1) | 51 (4.6) | 362 (8.2) | 546 (17.1) |  |
|  | Severe | 202 (2.3) | 8 (0.7) | 46 (1.0) | 148 (4.8) |  |
|  | Extreme | 14 (0.2) | 2 (0.2) | 4 (0.1) | 8 (0.3) |  |
| Usual activity | None | 522 (6.0) | 205 (18.4) | 245 (5.5) | 72 (2.3) | <.001 |
|  | Slight | 1496 (17.3) | 313 (28.0) | 838 (18.9) | 345 (11.2) |  |
|  | Moderate | 3156 (36.6) | 369 (33.1) | 1786 (40.3) | 1001 (32.5) |  |
|  | Severe | 2937 (34.0) | 208 (18.6) | 1372 (30.9) | 1357 (44.1) |  |
|  | Extreme | 519 (6.0) | 21 (1.9) | 196 (4.4) | 302 (9.8) |  |
| Pain/discomfort | None | 1080 (12.5) | 297 (26.6) | 647 (14.6) | 136 (4.4) | <.001 |
|  | Slight | 2263 (26.2) | 344 (30.8) | 1328 (29.9) | 591 (19.2) |  |
|  | Moderate | 3470 (40.2) | 335 (30.0) | 1734 (39.3) | 1392 (45.2) |  |
|  | Severe | 1625 (18.8) | 132 (11.8) | 669 (15.1) | 824 (26.8) |  |
|  | Extreme | 192 (2.2) | 8 (0.7) | 50 (1.1) | 134 (4.4) |  |
| Anxiety/depression | None | 3030 (35.1) | 607 (54.4) | 1615 (36.4) | 808 (26.3) | <.001 |
|  | Slight | 3035 (35.2) | 308 (27.6) | 1669 (37.6) | 1058 (34.4) |  |
|  | Moderate | 1882 (21.8) | 160 (14.3) | 912 (20.6) | 810 (26.3) |  |
|  | Severe | 580 (6.7) | 35 (3.1) | 215 (4.8) | 330 (10.7) |  |
|  | Extreme | 103 (1.2) | 6 (0.5) | 26 (0.6) | 71 (2.3) |  |

**Supplementary table 5.** Descriptive statistics EQ-VAS and EQ-5D utility score by phenotype

|  | | Cohort | Phenotype 1 | Phenotype 2 | Phenotype 3 | p-value |
| --- | --- | --- | --- | --- | --- | --- |
| EQ-VAS | Mean (SD) | 48.99 (18.55) | 59.03 (18.21) | 50.33 (17.62) | 43.40 (18.07) | <0.001 |
|  | Median (IQR) | 50 (27) | 60 (25) | 51 (25) | 40 (29) |  |
|  | Minimum | 0 | 0 | 0 | 0 |  |
|  | Maximum | 100 | 100 | 100 | 94 |  |
| EQ-5D utility score | Mean (SD) | 0.56 (0.26) | 0.69 (0.22) | 0.60 (0.23) | 0.45 (0.28) | <0.001 |
|  | Median (IQR) | 0.63 (0.34) | 0.74 (0.25) | 0.66 (0.26) | 0.50 (0.43) |  |
|  | Minimum | -0.41 | -0.18 | -0.33 | -0.41 |  |
|  | Maximum | 1 | 1 | 1 | 1 |  |
